# Third-Generation Cognitive and Behavioral Therapies for Obsessive-Compulsive Disorder: A Narrative Review

**DOI:** 10.7759/cureus.94299

**Published:** 2025-10-10

**Authors:** Pedro Veloso, Filipa Pereira

**Affiliations:** 1 Psychiatry, Hospital De Braga, Braga, PRT

**Keywords:** acceptance and commitment therapy, cognitive-behavioral therapy, metacognition, mindfulness, obsessive-compulsive disorder

## Abstract

Obsessive-compulsive disorder (OCD) is a chronic and disabling condition associated with significant distress and functional impairment. First-line treatments rely primarily on pharmacotherapy and cognitive-behavioral therapy (CBT), particularly exposure and response prevention (ERP). Although effective, a substantial proportion of patients continue to show clinically significant symptoms after treatment. In recent decades, alternative psychological therapies have been investigated to complement CBT and address its limitations in OCD management. This narrative review, based on the relevant literature indexed in PubMed/MEDLINE from 2003 to 2023, examines the evidence on third-generation therapies in OCD, with a focus on acceptance and commitment therapy (ACT), mindfulness-based therapy (MBT), and metacognitive therapy (MCT). Across study designs ranging from case reports to randomized controlled trials, these interventions have shown potential to reduce OCD symptoms, alleviate comorbid depression and anxiety, improve maladaptive beliefs, and enhance psychological flexibility. Despite promising findings, the evidence remains preliminary and constrained by small samples, heterogeneous designs, variable control conditions, and limited long-term data. Comparative studies with established first-line treatments are relatively few and show mixed findings, limiting conclusions about relative efficacy. Larger, well-controlled randomized trials with standardized outcome measures and long-term follow-up are needed to establish efficacy and clarify the role of third-generation therapies within OCD treatment algorithms.

## Introduction and background

Obsessive-compulsive disorder (OCD) is characterized by the presence of obsessions and/or compulsions. Obsessions are recurrent and persistent thoughts, images, or impulses that are experienced as intrusive and unwanted, typically causing significant anxiety. Compulsions are repetitive behaviors or mental acts that the individual feels driven to perform in response to an obsession or according to rigid rules, with the aim of reducing anxiety or distress, preventing a feared event, or achieving a sense of completeness [1,2]. For example, intrusive fears of contamination may lead to excessive handwashing, doubts about safety may result in repeated checking, and concerns about symmetry or order may lead to rituals such as arranging or counting. Current diagnostic classifications, namely the Diagnostic and Statistical Manual of Mental Disorders (DSM-5) and the International Classification of Diseases (ICD-11), highlight that obsessive-compulsive (OC) symptoms must be time-consuming (i.e., more than one hour per day) and associated with clinically significant distress or functional impairment [1,2]. Furthermore, OC symptoms must not be better explained by another mental disorder, or by the physiological effects of a substance or medical condition.

OCD is among the most common mental disorders in developed countries and ranks as one of the top 10 causes of disability worldwide [3]. Its lifetime prevalence in the general population is estimated at 2%-3%, typically emerging in adolescence or early adulthood, and, if untreated, tends to follow a chronic course [4]. It is often a disabling condition, leading to impairments in work, home management, and social functioning [4]. Moreover, OCD frequently co-occurs with other psychiatric disorders, most commonly anxiety, mood, impulse-control, and substance use disorders [4].

International treatment guidelines recommend selective serotonin reuptake inhibitors (SSRIs), clomipramine, and cognitive-behavioral therapy (CBT), particularly exposure and response prevention (ERP), as effective treatments for OCD [5,6]. CBT monotherapy is especially recommended as a first-line treatment for patients with mild to moderate OCD, in the absence of severe depression, provided the treatment is available and aligns with the patient’s preference. A combination of SSRIs and CBT is recommended for severe OCD, in the presence of comorbid conditions that may benefit from pharmacotherapy (e.g., major depression), or when monotherapy fails to achieve satisfactory results [7].

A meta-analysis found that psychotherapeutic interventions produced superior outcomes compared to pharmacological treatments [3]. However, most studies included patients on stable doses of antidepressants, leaving considerable uncertainty regarding their relative efficacy. Approximately half of patients with OCD do not respond fully to first-line treatments [8]. SSRIs are effective in only 40%-60% of patients and often fail to achieve full remission [9]. Although most patients experience significant improvement with CBT, a substantial proportion of patients continue to exhibit clinically significant OC symptoms after completing therapy. One study found that, following ERP treatment, 60% of patients recovered, defined as a Yale-Brown Obsessive Compulsive Scale (Y-BOCS) score of 14 or below, but only 25% became asymptomatic, defined as a Y-BOCS score of 7 or less [10]. 

Thus, current treatment options have limited efficacy, and there is room for improvement in psychological interventions for OCD. Alternative or complementary interventions have been proposed to address the limitations of CBT in the treatment of OCD.

This narrative review aims to provide a critical overview of the most widely studied third-generation approaches in OCD, namely acceptance and commitment therapy (ACT), mindfulness-based therapy (MBT), and metacognitive therapy (MCT). 

## Review

Methods

A PubMed/MEDLINE search was conducted covering the period between January 2003 and May 2023. Given the narrative (non-systematic) design of this review, the literature search was restricted to this database, which provides comprehensive coverage of clinical and empirical studies on OCD. Additional sources may nonetheless include relevant publications. The search strategy combined the terms “obsessive-compulsive disorder,” “third-generation therapies,” “third-wave therapies,” “acceptance and commitment therapy,” “mindfulness,” and “metacognitive therapy.”

The sources considered were empirical studies evaluating the clinical impact and effectiveness of these therapies in adult OCD populations, ranging from case reports and small series to uncontrolled and randomized clinical trials (RCTs). Conceptual and theoretical articles outlining the principles and clinical rationale of third-wave therapies were also reviewed, along with relevant systematic reviews, meta-analyses, and other key reference publications to provide theoretical and clinical background.

Exclusion criteria comprised qualitative studies, trial protocols, single-session or very brief interventions, mixed or poorly structured programs, ecological or momentary assessment studies, and non-systematic reviews. Studies in children or adolescents, in non-clinical samples, or restricted to OCD-related subtypes or spectrum disorders were also excluded. Only studies in English or Portuguese were considered.

Third-generation cognitive-behavioral therapy in obsessive-compulsive disorder

According to Hayes’ proposition, CBT can be classified into three generations. The first generation of behavioral therapy focuses on modifying dysfunctional behaviors, rather than thoughts and emotions, based on principles of conditioning and neobehaviorism [11]. The second generation emphasized the role of language and cognition in the development and treatment of psychopathology, particularly how maladaptive cognitions, such as automatic thoughts, beliefs, and cognitive distortions, contribute to emotional distress and dysfunctional behaviors [12,13].

Cognitive-behavioral models of OCD propose that obsessions stem from maladaptive interpretations of negative intrusive thoughts, which are commonly experienced in the general population [14-16]. Individuals with OCD tend to appraise these egodystonic cognitive intrusions as highly significant, threatening, and requiring control. These appraisals are thought to stem from dysfunctional core beliefs, such as an overestimation of threat, an inflated sense of personal responsibility, an exaggerated need to control thoughts, perfectionism, and intolerance of uncertainty [17]. Misinterpreting intrusive thoughts generates anxiety, leading individuals to engage in avoidance and compulsive rituals to alleviate the associated emotional distress [17]. These responses are counterproductive, as they elicit further intrusive thoughts and reinforce dysfunctional beliefs about their importance and danger, thus perpetuating a vicious cycle [17].

ERP involves gradually and repeatedly exposing individuals to stimuli that trigger obsessions and anxiety, followed by response prevention, that is, refraining from performing compulsive rituals. ERP promotes habituation to anxiety-provoking stimuli and tests whether the feared consequences occur. This process can reduce anxiety, decrease compulsive behaviors, and alter the interpretation of obsessions [18].

The third generation of CBT emerged in the 1990s. Third-wave approaches are particularly concerned with the context and function of psychological phenomena and behavior, rather than their mere form. Accordingly, they emphasize contextual and experiential strategies for promoting psychological change [11]. Rather than modifying the form, frequency, or situational sensitivity of negative thoughts and emotions as is typical in traditional CBT, third-wave approaches focus on altering the individual’s relationship with their internal experiences [13].

Examples of third-wave interventions include dialectical behavior therapy, mindfulness-based cognitive therapy (MBCT), ACT, MCT, and compassion-focused therapy, among others. These newer approaches have expanded into domains traditionally linked with less empirical clinical frameworks, emphasizing themes such as acceptance, mindfulness, dialectics, values, spirituality, and relational processes [11].

In recent decades, there has been growing interest in the potential role of third-wave therapies in the treatment of OCD, particularly ACT, MBT, and MCT. The following sections examine the distinctive features of these approaches and their specific applications in the treatment of OCD. 

Acceptance and Commitment Therapy

ACT is grounded in the philosophy of functional contextualism and in relational frame theory (RFT) [19]. Contextualism views psychological events as actions of an organism interacting within historically and situationally defined contexts. The core components of functional contextualism are: (1) a focus on the event as a whole, rejecting reductionism and considering events within their unique context; (2) sensitivity to the role of context in understanding the meaning and function of an event; (3) emphasis on a pragmatic truth criterion, in which what is considered true is defined by what works in practice; and (4) orientation toward specific goals, which guide the application of this pragmatic criterion [11]. According to RFT, the core of human language and cognition is a learned and contextually regulated ability to arbitrarily relate events [19]. These relational frames are characterized by mutuality (if A is related to B, then B is related to A), combinatoriality (if A is related to B and B to C, then A is related to C), and the capacity to transform the functions of events based on these relations [19]. In this framework, negative emotions may be verbally predicted, evaluated, and avoided, and stimulus functions derived from relational frames can dominate other sources of behavioral regulation, leading to reduced contact with the present moment and greater dominance of rigid rules and verbal evaluations [11].

According to ACT, the primary source of psychopathology is psychological inflexibility, that is, the inability to persist in or change behavior in the service of valued goals, and experiential avoidance (EA), defined as an unwillingness to engage with internal experiences, even when doing so causes psychological or behavioral harm [11,19]. EA is considered a core feature of OCD, as it entails resistance to, and efforts to escape from, distressing internal experiences such as obsessions. One study found that high levels of EA were positively associated with elevated levels of OCD symptoms [17]. Paradoxically, efforts to avoid aversive internal experiences may amplify their salience by reinforcing the underlying relational frames. This restricts behavioral flexibility, as potential actions may be perceived as triggering the feared experience [11,19]. 

ACT aims to enhance psychological flexibility, defined as the ability to contact the present moment and to persist or change behavior when doing so serves personally valued goals, as well as to foster committed action toward living a life in alignment with one's values.

Psychological flexibility is cultivated through six core processes: (1) acceptance (willingness to experience all internal events); (2) cognitive defusion (the ability to experience thoughts as separate from the self and not necessarily as guides for behavior); (3) present-moment awareness (flexibly attending to internal and external events as they occur, without judgment); (4) self-as-context (experiencing oneself as the context in which internal experiences occur, rather than being defined by them); (5) values (personally meaningful life directions); and (6) committed action (flexibly pursuing valued goals) [11,19]. Mindfulness exercises are widely used in ACT to promote present-moment awareness, self-as-context, and achieving cognitive defusion [11]. In addition to mindfulness practices, exposure exercises may also be employed within ACT, both serving the overarching aim of fostering psychological flexibility. In the context of OCD, reduced psychological flexibility contributes to rigid patterns of avoidance and compulsive rituals. By enhancing flexibility, ACT may help individuals relate differently to their obsessions and engage more fully in actions guided by personal values. 

The emphasis on personal values distinguishes ACT from other treatments. ACT encourages individuals to clarify their values across key life domains and to set meaningful, attainable goals that reflect those values, while working to overcome psychological and contextual barriers to action. Thus, ACT integrates both acceptance- and change-oriented strategies [11]. The application of ACT to OCD has been illustrated in case studies, nonconcurrent multiple-baseline single-case studies, and RCTs [20-28]. Case reports and single-case studies consistently reported reductions in OCD and affective symptoms, as well as increases in psychological flexibility, with gains usually maintained at follow-up [20-23]. 

Among RCTs, Twohig et al. found that ACT led to significantly greater reductions in OCD severity and depression, and higher clinical response rates, compared to progressive relaxation training, with gains maintained at follow-up. ACT participants showed greater short-term gains in psychological flexibility [24]. In contrast, another RCT showed that ACT combined with ERP and ERP alone both produced large and durable improvements in OCD symptoms, depression, and psychological flexibility, which were maintained at follow-up, with no significant differences between the two groups, indicating no incremental benefit of adding ACT to adequately delivered ERP [25]. In a three-arm trial, ACT and combined treatment (ACT+SSRIs) were more effective than SSRIs alone in improving OCD symptoms and reducing EA, with larger proportions achieving clinically significant change and remission, although no significant differences emerged between ACT and the combined treatment, demonstrating that adding SSRIs to ACT did not enhance short-term efficacy, possibly due to the latency period of pharmacological action [26]. Zemestani et al. found that both ACT+SSRIs and ERP+SSRIs were superior to SSRIs alone in reducing OCD severity and psychological inflexibility, with gains maintained at follow-up. The ACT group showed greater improvement in psychological flexibility compared to the ERP group; however, no statistical differences were observed between groups regarding OCD symptoms [27]. Finally, Zou et al. found that combining sertraline with either ACT or repetitive transcranial magnetic stimulation (rTMS) produced significant within-group improvements in OCD symptoms, depression, and anxiety, with no significant between-group differences in symptom severity, although ACT was superior in enhancing psychological flexibility [28].

A systematic review and meta-analysis by Soondrum et al. confirmed that ACT significantly reduces OCD severity compared to controls, with a large overall effect. ACT was especially effective against inactive controls, comparable to active comparators such as ERP or SSRIs, and consistently improved psychological flexibility [29].

Overall, these findings indicate that ACT reduces OCD symptoms, improves comorbid depression and anxiety, and enhances psychological flexibility [20-28]. In sum, evidence suggests ACT is an efficacious treatment for OCD, superior to progressive relaxation training and SSRIs, and comparable to ERP on clinical outcomes, being particularly valuable for patients unwilling or unable to engage in ERP. As an adjunct to medication, some evidence suggests ACT may provide added benefit, although as an adjunct to ERP, no incremental advantage has been demonstrated.

Despite these promising results, methodological limitations such as small sample sizes, heterogeneous designs, variable control conditions, and short follow-up periods complicate cross-study comparisons and restrict conclusions on long-term efficacy.

Mindfulness-Based Therapy

Rooted in Buddhist meditation traditions, mindfulness has gained prominence in Western medicine and psychology largely due to the work of Jon Kabat-Zinn, who introduced it in a secular clinical framework and developed the mindfulness-based stress reduction (MBSR) program in 1979. Over the past two decades, MBTs have expanded to include structured group programs such as MBSR and MBCT, and are now widely applied across a range of psychological problems, with evidence supporting their efficacy in reducing symptoms of anxiety and depression [30]. Despite the growing evidence base, the role of MBT in the treatment of OCD remains less clearly defined.

Although the precise definition of mindfulness remains debated, it is commonly described, following Kabat-Zinn, as “the awareness that emerges by paying attention, on purpose, in the present moment, and non-judgmentally to the unfolding of experience, moment by moment” [31]. It comprises three interrelated components: (1) self-regulation of attention; (2) an attitude of acceptance, compassion, curiosity, and openness; and (3) present-moment awareness [32]. 

MBT encourages non-judgmental awareness and acceptance of thoughts, bodily sensations, emotions, and impulses. In the context of OCD, this may help reduce distress associated with obsessions, promote habituation to intrusive thoughts, and decrease avoidance and compulsive behaviors [33]. Mindfulness also cultivates non-reactivity, the ability to allow cognitive experiences to arise and pass without engaging with or attempting to suppress them. Patients are encouraged to notice and acknowledge their thoughts without judgment and to refrain from acting on them compulsively.

MBT may support OCD treatment by: (1) enhancing engagement with ERP through a non-avoidant and non-ruminative stance; (2) improving insight, reality testing, and acceptance of the transient nature of thoughts, emotions, and sensations; (3) promoting metacognitive awareness and cognitive defusion; (4) increasing self-efficacy, self-confidence, and self-compassion; and (5) addressing cognitive and metacognitive biases [34]. Thus, MBT may help individuals develop a more adaptive relationship with their obsessions and reduce their reliance on compulsions.

The potential of MBT for treating OCD has been explored in case series, open-label uncontrolled trials, and RCTs [33-40]. Preliminary uncontrolled studies consistently reported reductions in OCD symptoms, comorbid depression and anxiety, and dysfunctional obsessive beliefs, alongside improvements in mindfulness skills and quality of life [35-37]. 

Two RCTs support the utility of MBT in individuals with residual OC symptoms following completion of CBT, showing additional symptom relief [33,38]. Key et al. showed that MBCT led to significantly greater improvements in OCD symptoms, depression, anxiety, obsessive beliefs, and mindfulness skills compared to a waitlist control [33]. Külz et al. reported no between-group differences on clinician-rated OCD severity, but MBCT showed short-term advantages compared to psychoeducation on self-reported OC symptoms, depression, obsessive beliefs, mindfulness skills, and quality of life; however, these effects were not maintained at follow-up [38]. Extending the follow-up, Cludius et al. observed sustained reductions in OCD symptoms and secondary outcomes at 12 months in both MBCT and psychoeducation groups, without significant between-group differences [39]. Zhang et al. found comparable efficacy between MBCT and SSRIs in unmedicated patients with OCD, with both superior to psychoeducation in responder rates at post-treatment, although this advantage was not sustained at follow-up, and Y-BOCS reductions did not differ between groups. Although average symptom reductions were comparable across groups, a greater proportion of patients in MBCT and SSRIs achieved clinically meaningful response thresholds. For depression and anxiety, differential changes between groups were indicated, though the specific group contrasts were not reported. Increases in mindfulness were observed across all groups without significant between-group differences [34]. Mathur et al. reported MBCT to be superior to stress management training, with significantly higher responder rates, greater reductions in OCD severity, obsessive beliefs, and anxiety, though no differences were reported regarding depression, mindfulness skills, or quality of life [40].

A systematic review and meta-analysis by Riquelme-Marín et al. found that mindfulness-based interventions produced medium-sized improvements in OCD symptoms, with smaller effects for depressive symptoms and mindfulness coping. These benefits were observed both as stand-alone treatments and when used as adjunctive interventions following CBT [41].

Taken together, the evidence indicates that mindfulness-based interventions are associated with meaningful reductions in OCD symptoms, comorbid depression and anxiety, and dysfunctional obsessive beliefs, as well as improvements in mindfulness skills [33-40]. While some RCTs show MBCT to be comparable to first-line pharmacotherapy and superior to psychoeducation or stress management, results are not entirely consistent, and between-group differences often diminish at follow-up, suggesting accelerated but not always sustained benefits.

Several limitations temper these findings, including small-to-moderate sample sizes, heterogeneous study designs, and variable control conditions, which complicate comparisons, and scarce long-term data, which further constrain interpretation.

Metacognitive Therapy

Metacognition, a concept closely related to mindfulness and cognitive defusion, refers to the cognitive processes that monitor, regulate, and appraise thinking itself [42]. The metacognitive model of OCD proposes that negative thoughts and emotions are naturally transient but may persist and escalate into psychological difficulties when individuals adopt maladaptive cognitive styles that disrupt self-regulation [42-44]. This pattern, known as the cognitive attentional syndrome (CAS), includes worry, rumination, threat monitoring, and maladaptive coping strategies such as compulsions [12]. 

Two core metacognitive domains have been identified in OCD: (1) beliefs about the meaning and power of thoughts (fusion beliefs) and (2) beliefs about rituals [44]. Three main types of fusion beliefs have been described: (1) thought-event fusion, the belief that a thought can cause an event or implies that the event has already occurred (e.g., “having an intrusive image of running someone over means I must have done it”); (2) thought-action fusion, the belief that a thought will lead to action or is morally equivalent to it (e.g., “thinking about hurting someone means I will do it”); and (3) thought-object fusion, the belief that thoughts or feelings can contaminate or be transferred to objects (e.g., “bad thoughts can contaminate objects”). Beliefs about rituals involve two components: (1) beliefs regarding the necessity of performing rituals (e.g., “I have to wash my hands, or I will never feel okay”) and (2) internal rules or stop signals, that is, subjective criteria that guide when a ritual can be discontinued (e.g., “I must check until I feel completely certain”).

MCT aims to modify dysfunctional metacognitive beliefs to enhance control over cognitive processes. Detached mindfulness (DM), a central technique in MCT, encourages individuals to adopt a new relationship with intrusive thoughts by fostering metaconsciousness, a state in which the self and cognitive events are experienced as separate from one another [42,43]. In this way, thoughts are observed as transient mental events rather than as dangerous or true, allowing intrusive thoughts to be experienced without the need to engage in maladaptive responses.

Empirical studiessupport the metacognitive model by showing that reductions in OCD symptoms are more strongly predicted by changes in metacognitive beliefs (e.g., positive beliefs about worry, negative beliefs about uncontrollability/danger of thoughts and the need to control thoughts, thought-fusion beliefs, beliefs about rituals, and stop-signal beliefs) than by modifications in traditional cognitive beliefs related to responsibility or perfectionism [45-48]. Additionally, Park et al. found that metacognitive beliefs also predicted pharmacological outcomes; patients with lower baseline positive beliefs about worry responded more rapidly to SSRIs [49]. This suggests that maladaptive metacognitions may function as negative prognostic markers for pharmacological response, highlighting the potential clinical value of targeting them alongside medication.

Research on MCT in OCD remains limited and consists of a case report, a case series, an open-label uncontrolled trial, a nonconcurrent multiple-baseline single-case study, a naturalistic cohort study (benchmarking/clinical audit)+, and two RCTs [50-56]. Preliminary case reports and small case series documented that MCT can lead to dramatic reductions in OCD severity, in some cases to asymptomatic levels, alongside marked reductions in depression, anxiety, and dysfunctional metacognitive beliefs [50,51]. Open and uncontrolled trials also found large, statistically significant pre-post improvements in OCD severity, with most participants achieving clinically significant change, and parallel reductions in dysfunctional metacognitive beliefs and depressive symptoms, with effects maintained at follow-up [52,53]. 

In routine clinical care, Papageorgiou et al. found that both group CBT and group MCT significantly reduced OCD severity, depression, and functional impairment. While post-treatment scores were similar, MCT patients demonstrated significantly higher response rates [54]. 

Randomized evidence remains limited. In the trial by Rupp et al., both detached mindfulness (a metacognitive strategy) and cognitive restructuring produced large and significant improvements in OCD severity compared to waitlist, with large effect sizes and approximately 40% of patients achieving clinically significant change. No differences emerged between the two active interventions [55]. Hansmeier et al. compared ERP and MCT in a small pilot RCT, reporting large pre-post reductions in OCD severity in both groups; however, inferential tests were not reported to determine whether one treatment outperformed the other on OCD symptom reduction. At the metacognitive level, MCT was superior in reducing thought-fusion beliefs. The study also found that reductions in ritual- and stop-signal-related beliefs predicted subsequent improvements in OCD symptoms, supporting the role of metacognitive change as a key mechanism of therapeutic response [56].

A systematic review by Philipp et al. found evidence only for self-help metacognitive training (myMCT), which showed modest benefits over standard treatment, but high rates of partial non-adherence [57]. No therapist-delivered MCT trials were available at that time, underscoring how the present evidence extends the field by demonstrating potentially larger and clinically meaningful benefits of MCT when applied as a structured psychotherapy.

Overall, the emerging evidence suggests that MCT can produce large and clinically meaningful improvements in OCD symptoms, depressive symptoms, and dysfunctional metacognitive beliefs [50-56]. Evidence also highlights the central role of metacognitive change in mediating treatment response.

Nevertheless, most studies are limited by small samples, uncontrolled designs, scarce RCTs, variable control conditions, and short follow-up, which complicates synthesis and limits comparability across studies.

## Conclusions

Third-generation therapies offer innovative approaches to treating OCD by focusing on how individuals relate to their internal experiences, emphasizing acceptance, mindfulness, and metacognitive processes. Preliminary evidence suggests these interventions may reduce OCD symptoms, alleviate comorbid depression and anxiety, improve maladaptive beliefs, and enhance psychological flexibility.

Despite promising findings, current evidence remains preliminary, constrained by small samples, methodological heterogeneity, scarce long-term data, and a limited number of comparative studies with established first-line treatments. Future research should prioritize large, well-controlled randomized trials with standardized outcome measures and extended follow-up to establish efficacy and clarify the role of third-generation therapies within OCD treatment algorithms. In addition to clarifying efficacy, the successful integration of these therapies into clinical practice will require investment in practitioner training and improved accessibility within mental health services, thereby expanding therapeutic options and enabling more personalized care for patients who do not fully respond to standard treatments.
